# Cerebrospinal Fluid and Blood Biomarkers of Neuroaxonal Damage in Multiple Sclerosis

**DOI:** 10.1155/2011/767083

**Published:** 2011-05-02

**Authors:** Irena Dujmovic

**Affiliations:** Neurology Clinic, Clinical Center of Serbia, Dr Subotica 6, P.O. Box 12, 11129 Belgrade 102, Serbia

## Abstract

Following emerging evidence that neurodegenerative processes in multiple sclerosis (MS) are present from its early stages, an intensive scientific interest has been directed to biomarkers of neuro-axonal damage in body fluids of MS patients. Recent research has introduced new candidate biomarkers but also elucidated pathogenetic and clinical relevance of the well-known ones. This paper reviews the existing data on blood and cerebrospinal fluid biomarkers of neuroaxonal damage in MS and highlights their relation to clinical parameters, as well as their potential predictive value to estimate future disease course, disability, and treatment response. Strategies for future research in this field are suggested.

## 1. Introduction

Multiple sclerosis (MS) is a chronic disease of the central nervous system (CNS) characterized by unpredictable clinical relapses and remissions and/or by progression of disability over time [1]. Relapses are considered to be the clinical expression of acute inflammation in the CNS, whereas progression reflects chronic demyelination, gliosis, and axonal loss [2]. Although axonal/neuronal damage has been recognized in MS for more than a century [3], a refocused interest on the role of axonal pathology and neurodegeneration as the cause of permanent neurological disability in MS patients appeared since the 1990s [4–9]. The development of new immunostaining protocols and new magnetic resonance imaging (MRI) techniques has enabled earlier detection of more subtle changes in diffuse neuroaxonal pathology not only within focal white matter [6, 10] and gray matter lesions [11–13], but also within normal appearing white matter (NAWM) [14–16] and normal appearing gray matter in MS [14, 15]. Current evidence suggests that axonal loss occurs at an early stage of MS [6, 17], but because of CNS compensatory mechanisms it remains clinically silent until a threshold level of axonal loss is achieved and the functional reserve capacity is exhausted [9, 18]. Subsequent progressive axonal loss underlies a continuous and irreversible neurological decline [19], causing a transition from initially relapsing remitting (RR) to the secondary-progressive (SP) MS [7, 9]. 

Since inflammation correlates only poorly with disability and the loss of neurons and axons may be subject to biochemical monitoring [20], biochemical markers of neuroaxonal degeneration gain increasing importance. Such biomarkers could provide tools for development and evaluation of new therapeutic strategies [21] and might serve as prescreening tools and/or cross-sectional surrogate endpoints in MS clinical trials [22, 23], more importantly in those testing potentially axon-protective compounds [24]. Additionally, the assessment of neuroaxonal biomarkers could help in better understanding of MS pathogenesis and identification of disease subtypes [22], as well as in routine patient management for (1) prediction of conversion to MS after a first clinical episode, (2) early prediction of disease course and future disability, (3) selection of patients for individually tailored treatments, and (4) monitoring of disease activity and individual treatment response [23, 25–27]. However, it is unlikely that a single biomarker could serve for any of these aims due to the extreme complexity of the pathogenetic processes which cause tissue damage and neuroaxonal loss in MS [26].

Recent research has introduced new candidate biomarkers but also elucidated pathogenetic and clinical relevance of the well-known ones. This paper reviews the existing data on blood and cerebrospinal fluid (CSF) biomarkers of neuroaxonal damage in MS in the light of their clinical relevance and suggests strategies for future research in this field.

## 2. Mechanisms of Neuroaxonal Damage in MS

**Figure 1:**
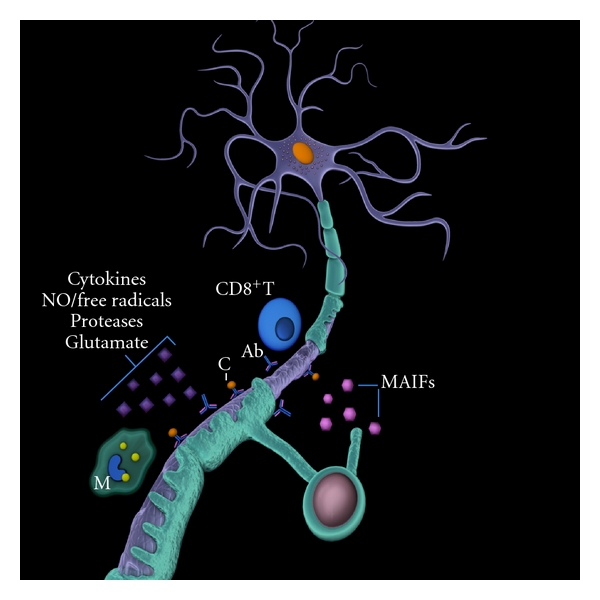
The mechanisms of neuroaxonal damage in multiple sclerosis. Legend: NO: nitric oxide; M: macrophage; C: complement; Ab: antibody; CD8^+^T: CD8^+^T-lymphocyte; MAIFs: myelin-associated inhibitory factors.

The mechanisms leading to axonal damage in MS are essentially not well elucidated [21]. However, challenging some clinical [28–30], neuroradiological [31], and neuropathological [11, 32] observations that neurodegeneration in MS might progress independently from or even precede the inflammation, recent neuropathological reports confirmed the positive correlation between axonal pathology and the degree of inflammation even in cases with progressive MS [33, 34]. This further supports the scenario in which a variety of effectors from the inflammatory microenvironment could injure axons, such as direct attack by autoreactive antibodies [35–37] or cytotoxic CD8^+^T-cells [36, 38, 39], invading macrophages, proteolytic enzymes, cytokines, nitric oxide [39–41] and free radicals [42, 43], defects in calcium homeostasis [21], glutamate-mediated excitotoxicity [43, 44], an increased axonal energy demand [45], and mitochondrial injury and failure [45, 46] (Figure 1). Axonal damage could also be secondary to acute or chronic demyelination [6, 47], damaging changes in sodium channel distribution [2, 48], and disruption of axonal/glial interactions [49, 50] as well as related to the lack of myelin-derived trophic deprivation and/or impaired axonal regeneration by axon growth inhibitory molecules including those from myelin debris recently called myelin-associated inhibitory factors (MAIFs) such as myelin-associated glycoprotein, oligodendrocyte myelin glycoprotein, Nogo-A, semaphorin 4D/CD100, and ephrin B3 [51, 52] (Figure 1).

## 3. Biomarkers of Neuroaxonal Damage in Multiple Sclerosis

**Figure 2:**
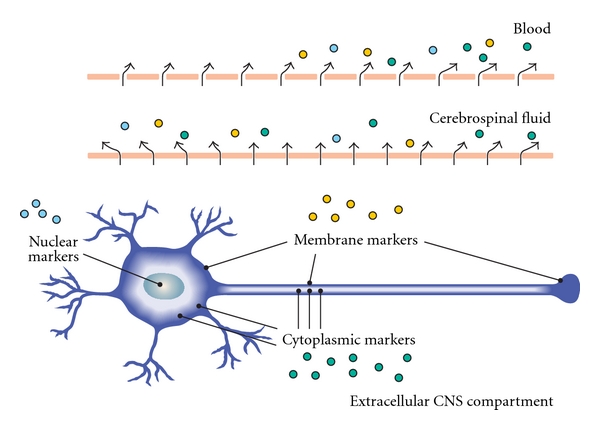
Membrane, cytoplasmic, and nuclear markers of neuroaxonal damage in the central nervous system (CNS) and their dynamics within three compartments (extracellular space, cerebrospinal fluid, and blood).

Following damaging processes, molecules released from neuronal cytoplasm, membrane, or nucleus are released into the extracellular CNS compartment (Figure 2) and their further metabolic, transport, and reuptake mechanisms, drainage pathways or other interactions with the CNS tissue, as well as the degree of tissue destruction would determine the level of these substances in CSF and blood [21]. 

CSF analysis is more pathology specific as it provides information from the body fluid that is most closely associated to the disease process [53], but sometimes substances measured in lumbar-sac-CSF are not necessarily completely representative of brain pathology [20]. However, there is a need for new biomarkers in more easily accessible body fluids such as peripheral blood [53], since substances produced within the CNS and found in the blood could also be representative of the ongoing CNS pathology [20]. 

CSF and/or blood levels of biomarkers associated with neuroaxonal injury in patients with MS and clinically isolated syndrome suggestive of MS (CIS) are summarized in Tables 1, 3, 4 and 5 and their relation to clinical parameters in Table 2.

### 3.1. Neurofilaments

Neurofilaments (NFs) are cytoskeletal proteins which play a role in stabilizing axons, determining axon diameter and participate in axonal transport [54, 55]. As NFs are found exclusively within neurons, their detection in blood or CSF therefore reflects neuronal and axonal damage [56]. Mammalian NFs consist of different subunits: NF-light chain (NF-L), which serves as a backbone for NF- intermediate chain (NF-M) and a heavy chain (NF-H) to copolymerise [21]. The most abundant, the smallest and most soluble NF subunit is NF-L but is susceptible to proteases [57]. On the other hand, HF-H is a larger molecule and more resistant to proteases if phosphorylated [58]. Phosphorylated parts of NF molecules are mostly abundant within HF-M and NF-H subunits [59], and the state of phosphorylation influences axonal diameter [60]. The highly phosphorylated NF-H are normally found only in axonal NFs and this marker is thought to indicate axonal injury and/or degeneration [56], whereas NF-L constitutes only a minor part of the neuronal cell body and dendrites relative to axons [61]. NF-H phosphorylation may increase during the progressive phase of MS [62]. Due to the lower molecular weight of NF-L and/or its lower phosphorylation rate, NF-L could diffuse earlier from the parenchyma into CSF than NF-H, but also could be degraded quicker [54]. Although changes in the blood-brain barrier (BBB) might influence the CSF NF-L concentration, the degree of such influence was considered to be negligible [61].

Healthy individuals have no NF-L in their CSF, whereas most people with neurological disorders, such as amyotrophic lateral sclerosis, stroke, MS and Alzheimer's disease, can have elevated levels [73]. Several studies have shown the increase of the CSF NF-L levels in MS or CIS patients (Table 1), in the latter more so in those who converted to MS within 3 years [54]. On the other hand, CSF NF-L was detectable at low concentrations [74], or even undetectable in some other studies albeit the assay was similar to that used by others [66]. CSF NF-L levels were reported to be increased during acute relapses [54, 64], in patients with enhancing MRI lesions [54], as well as in patients with higher relapse rate [61] (Table 2) and were also shown to have a peak during the first two months after the start of the previous exacerbation and to gradually decrease thereafter to a low level [61]. A correlation between NF-L with Expanded Disability Status Scale (EDSS) score as a disability measure was found in some studies [54, 63]. Norgren et al. [65] reported a significant correlation between CSF NF-L levels and progression index over 10 years whereas in a recent study the risk for high *Multiple Sclerosis* Severity Score (*MSSS*) at long-term follow-up after 14 years was increased threefold for cases with high NF-L levels [75]. Conversion from RRMS to SPMS was more likely in cases with high CSF NF-L levels when compared with those with undetectable or intermediate NF-L levels [75] (Table 2). Other authors could not demonstrate any correlation with disability measures [64, 66]. In some studies CSF NF-L concentration did not correlate with gender or age [61, 64, 66, 67] or disease duration [64, 65], but in some reports CSF NF-L levels were found to increase with age [68]. Blood NF-L levels have not been reported to date (Table 1).

NF-M subunit has not been analysed so far in body fluids of MS patients.

In patients with optic neuritis (ON), the levels of NF-H phosphorylated form (NF-H^SM135^) in plasma [71], or its hyperphosphorylated form in CSF (NF-H^SM134^) [69], as well as CSF NF-H^SM135^ levels in CIS patients [25], were found to be significantly higher compared to controls (Table 1). Significantly higher CSF NF-H levels in MS patients than in control subjects (Table 1) were also reported in several recent studies (Table 1) [23, 54, 72], with higher [70], or significantly higher [54, 62] levels in patients with a progressive course. Opposite to these findings, no difference in CSF levels of this biomarker was found between RR, SP and primary progressive (PP) MS patients by Eikelenboom et al. [66]. In some studies, CSF NF-H levels correlated significantly with EDSS score both in CIS [25] and MS patients [54, 62]. CSF NF-H levels also significantly correlated with the ambulation index and the nine-hole-peg test scores [62], as well as with the MSSS [76]. In the latter study the degree of NF phosphorylation (ratio, hyperphosphorylated versus phosphorylated NF-H) was 8-fold higher in severely versus mildly disabled patients [76], whereas no correlation of NF-H levels with EDSS was found in some other studies in CSF [66] or plasma [77]. The highest CSF NF-H levels were found during relapses [25, 54] or correlated with MRI lesion enhancement [78], but Petzold et al. found no correlation of plasma HF-H^SM135^ with the relapse rate [77]. In the study of Brettschneider et al., the sensitivity for predicting conversion to clinically definite (CD) MS after CIS was generally low, but could be increased by combining MRI with CSF NF-H criteria [25]. Additionally, a tendency towards a higher RRMS to SPMS conversion rate over 3 years in patients with high CSF NF-H levels was also shown [62]. Moreover, Petzold et al. [71] found significantly higher plasma NF-H levels in ON patients with poor recovery of visual acuity than in those with good recovery. In the study of Lim et al., in which 8/18 patients in the ON trial and 15/32 subjects in the MS attack trial were treated with oral methylprednisolone, in the MS attack trial group, CSF NF-H^SM134^ and NF-H^SM135^ measured at week 3 and CSF NF-H^SM134^levels from baseline to week 3 were predictive of clinical outcome at week 8 and 52 [78]. In the study of Rejdak et al., CSF NF-H levels inversely correlated with the EDSS recovery grade over a short-term follow-up of 6–8 weeks [72]. Moreover, in 30 RRMS patients, plasma NF-H^SMI35^ levels were higher, albeit nonsignificantly, in nonresponders than in responders to IFN beta1-a or 1-b over 1 year of treatment [77]. A correlation of CSF NF-H levels with age was found in CIS or MS patients in some studies [54], but in the others no age influence [62, 66] or a correlation with disease duration was found [66, 72].

### 3.2. Antineurofilament Antibodies

Axonal damage and subsequent exposure of NFs could lead to antibody generation in a T-cell-dependent secondary immune response to a foreign antigen [85]. Cytoskeletal and myelin debris, released by neurons, are removed by macrophages which may be able to reach the peripheral lymph nodes [86]. Additionally, anti-NF-antibodies could be induced from exposure to exogenous agents, possibly virus-derived peptides and subseqently may cross-react with neuronal antigens [87].

Autoimmune responses to neuronal antigens might contribute to axonal damage and irreversible disability in MS [12], but could also be an epiphenomenon [79]. In the latter study, intrathecal immunoglobulin (Ig) G and IgM anti-NF-L synthesis did not differ between MS subgroups (RR, SP, or PP) or between CIS, MS patients, or healthy controls [79] (Table 3). On the other hand, the intrathecal anti-NF-L IgG was shown to correlate with MRI parameters of cerebral atrophy [66] and NF-L-autoimmunity has been also recently reported to be pathogenic in mice [12]. Additionally, anti-NF-L-IgG levels in serum were found to be significantly increased in PPMS patients compared to other neurological diseases or healthy controls [80] and in some studies a specific CSF/serum anti-NF-L IgG index correlated with EDSS or MSSS scores [74, 81] (Table 2). In some reports, anti-NF-L levels did not correlate with age or disease duration or EDSS score [66, 80], although some other authors showed a correlation between both anti-NF-L IgG index and CSF anti-NF-L IgG and duration of symptoms before lumbar puncture [74]. 

Anti-NF-M antibody response was analyzed in MS in a few studies. In a study of Bartoš et al., the extent of anti-NF-M antibody levels did not correspond to any individualized clinical profiles of MS patients although the intrathecal production of IgM and IgG anti-NF-M was significantly increased in all MS subgroups compared to patients with other diseases or healthy controls [82] (Table 3). In the latter study [82], the intrathecal IgG and IgM anti-NF-M synthesis in MS patients was unrelated to gender, age, disease duration, and EDSS score, but Fialová et al. [81] found a correlation between anti-NF-M IgG intrathecal synthesis and disability. 

Anti-NF-H IgG in CSF/serum was found to be similar in MS patients and controls [74] and between RR, SP, and PPMS patients [66] (Table 3). The intrathecal production of anti-NF-H IgG correlated with some parameters of brain atrophy such as the parenchymal fraction [74]. The CSF anti-NF-H levels correlated with the duration of disease before lumbar puncture and EDSS score in the study of Silber et al. [74], but no correlation with EDSS was shown by others [66].

### 3.3. Tubulin and Antitubulin Antibodies

In addition to NFs, the other major component of the axonal cytoskeleton is the microtubule, which mainly consists of *α* and *β* tubulin subunits [21], but also of microtubule-associated proteins (MAPs) such as MAP2 and tau [88]. Tubulin comprises as much as 20% of the cellular protein in brain [89] and is mainly responsible for axonal migration and longitudinal growth as well as for providing the conduit for fast axonal transport [90]. CSF tubulin levels were found to be increased in progressive MS (SP+PP) patients as compared to RRMS or controls [63] (Table 4). Antitubulin antibodies were also investigated and in some studies CSF levels of these antibodies were shown to be increased in MS patients [83], a finding which was not shown by others [74]. CSF tubulin levels [63], but also CSF antibodies to tubulin and the CSF/serum antitubulin index correlated significantly with EDSS in one study [74], whereas no similar correlations with disability were found in two other studies [81, 83] (Table 2). No correlations of antitubulin antibodies were found with age or disease duration [74, 83].

### 3.4. Actin

Actin is the major component of the microfilaments [21]. CSF actin levels were found to be significantly elevated in MS patients than in the control group, with higher levels in progressive MS cases and correlated with the EDSS scores [63] (Table 4). Anti-actin antibodies were not separately investigated in MS patients to date and could also be detected in normal sera [91].

### 3.5. Gelsolin

Gelsolin is an actin-binding protein that regulates actin organization [92] and is expressed in neurons in addition to the other cells [93]. Additionally, its secreted isoform could be found in the circulation [94] and belongs to the extracellular actin scavenger system [95]. Following some preliminary results showing low blood and CSF gelsolin concentration in 4 MS patients [92], Kulakowska et al. recently reported significantly lower plasma gelsolin levels in MS samples than in controls, whereas there was no difference in its CSF levels between the two groups [96] (Table 4).

### 3.6. Tau Protein

Tau is an axonal cytoskeletal protein that is involved in microtubule assembly and stabilisation [97] and therefore is essential for the efficient axonal transport [98]. Abnormal phosphorylation of tau can lead to the formation of potentially neurotoxic insoluble tau aggregates that have been shown to be characteristic features of common neurodegenerative diseases [97, 99, 100]. Pathological studies demonstrated the association of abnormally phosphorylated tau (p-tau) protein with SPMS and PPMS [101, 102] but also the absence of insoluble tau fraction in early MS, thus indicating the possibility that insoluble tau accumulates with disease progression [100, 102].

Total-tau (t-tau) protein and/or p-tau have been investigated in a respectable number of studies performed in CIS [25, 54, 103–106] and MS patients (Table 4) and the reported results are quite contradictory. Tau protein could be detectable in serum, but in concentrations that are ten times lower than in CSF [21]. CSF t-tau levels in CIS have been reported to be higher than in controls [25], but other authors found no difference in t-tau or p-tau compared to controls [104–106] (Table 4). In some MS studies, CSF levels of t-tau [103, 107–110] and p-tau [109] were reported to be significantly higher in MS patients than in controls, whereas some other authors could not confirm these differences [54, 104, 111]. T-tau and p-tau were also investigated in childhood RRMS cases in which their CSF levels were similar to controls [112]. A positive correlation of CSF t-tau levels was shown with EDSS in RRMS and CIS patients [25] and with the progression index over 3 years in early RRMS [113], but in some other studies no correlation with disability in CIS or MS was demonstrated for the CSF [103, 104, 110] or serum and CSF levels [105] (Table 2). Two studies have indicated an increased CSF-tau release in clinically active disease states [109, 114], in one study there was a significant elevation of CSF t-tau among patients with gadolinium-enhancing brain MRI lesions [103], but the latter finding was not confirmed afterwards [25]. However, the relation of CSF tau levels with the extent of intrathecal inflammation in MS was also supported by findings of Bartosik-Psujek and Archelos who showed a significant positive correlation between CSF t-tau levels and IgG index [108]. As shown by Brettschneider et al., the sensitivity and specificity of CSF tau levels for predicting CIS conversion to CDMS was generally low, but could be increased by combining with MRI parameters or with NF-H^SM135^ levels [25]. Gajofatto et al. [115] could not show a significant predictive value of CSF t-tau in patients with acute myelitis either for conversion to MS or for disability after a median followup of 6.2 years, but in the study of Martínez-Yélamos et al. [113], CSF-t-tau was the only independent variable to predict time to the next relapse (Table 2). Interestingly, phosphorylation of tau and axonal pathology were significantly reduced when EAE rats were treated with prednisolone [116], but similar findings in MS were not reported. Although CSF t-tau levels were found associated with age in the control group in the study of Bartosik-Psujek and Archelos [108], no correlation of CSF t-tau/p-tau levels with age or disease duration was found in the majority of other studies [104, 106, 107, 110, 111, 117].

### 3.7. Amyloid-Precursor Protein and Related Molecules

Amyloid precursor protein (APP) probably works as a cargo receptor for binding proteins during axonal transport [21], but could have some other important neural functions including those in memory processes [118]. APP is transported by a fast anterograde axonal transport [119] and subtle changes in axonal transport or axonal transection could lead to APP deposits in the axon that are easily detectable by immunocytochemistry [120]. Based on immunopathological findings, it was suggested that APP accumulation could be a sensitive marker of MS disease progression [121], but also a potential marker of acute or active MS [10]. APP is cleaved by an integral membrane aspartyl protease (*β*-site APP-cleaving enzyme 1, BACE1), which results in the release of N-terminal *β*-cleaved soluble APP (*β*-sAPP). The C-terminal fragment is further processed by *γ*-secretase to yield the amyloid beta (A*β*42) and the APP intracellular domain [122]. APP can also undergo *α*-secretase-mediated cleavage, which results in the release of the solubile *α*-sAPP [123]. 

In the recent study of Mattsson et al. [122], CSF levels of *α*-sAPP, *β*-sAPP, and A*β*42 were significantly lower in MS patients than in controls and patients with ongoing or recent MS exacerbation had lower *α*-sAPP levels than stable MS patients. CSF BACE1 activity was slightly reduced in patients with MS compared to controls and patients with SPMS tended to have lower BACE1 activity than patients with RRMS [122]. Baseline BACE1 activity did not predict change in EDSS score after 10 years (Table 2) but low BACE1 activity was associated with prolonged MS disease duration and disease severity. In contrast to controls, a reduction in BACE1 activity over 10 years was seen only in RRMS, whereas patients with SPMS displayed constantly low BACE1 activity levels [122]. Additionally, two molecules, Bri2 and Bri2-23 have been shown to interact with APP and regulate A*β*42 cleavage and aggregation *in vivo* [124, 125]. Recent findings revealed that CSF levels of Bri2-23, a peptide cleaved from Bri2, may be a biomarker of cerebellar/cognition dysfunction in progressive MS patients in which CSF Bri2-23 levels have been recently shown to be decreased [125]. In a recent study of Mori et al. [126], CSF A*β*42 levels were significantly lower in cognitively impaired MS patients and were inversely correlated with MRI parameters of disease activity. On the contrary, Vališ et al. [106] found a significantly higher CSF A*β*42 levels in MS patients compared to the control group (Table 4). No study has followed the effect of treatment on CSF-APP-derived proteins although some preliminary results in 16 nondemented, non-MS patients indicated a significant decrease in CSF A*β*42 levels following corticosteroid treatment [127]. CSF A*β*42 levels were not shown to correlate with age or disease duration [122, 126].

### 3.8. N-Acetylaspartate

N-Acetylaspartate (NAA) is the amino acid synthesized and almost exclusively localized in neurons [128] and is one of the most abundant molecules in the CNS [129]. Postmortem studies of spinal cords from MS patients related lower tissue concentrations of NAA to the lower axonal volume [8]. Several functions of NAA molecule in the CNS have been hypothesized, such as (a) an osmolite to remove water from neurons, (b) an acetate contributor in myelin sheath synthesis, (c) a mitochondrial energy source, (d) a precursor for *N*-acetylaspartyl glutamate, and (e) a ligand for certain metabotropic glutamate receptors [130]. Brain proton MR spectroscopy (MRS) allows *in vivo* examination of axonal integrity by quantifying the resonance intensity of NAA [131]. Previous proton MRS studies have found the reduced NAA levels in MS lesions, the surrounding NAWM and cortical grey matter [132], and a decline in global MRS-NAA levels was also demonstrated in benign MS [133]. A decrease in relative NAA levels by proton MRS was found in patients with CIS in CNS gray matter [134] and WM lesions [135]. Other studies have shown that NAA decrease in lesions and NAWM is related to clinical disability and progressive brain atrophy [136–138] and was indicated to be a feature of progression [16]. Moreover, some preliminary MRS findings have shown a beneficial effect of glatiramer acetate on increase of relative NAA peaks in MS lesions and NAWM over 2 years [131], whereas relative NAA peaks had become significantly higher in the interferon beta-lb -treated MS patients following 1 year of treatment [139]. 

In the study of Jasperse et al. [141], CSF concentrations of NAA correlated with EDSS and MS Functional Composite (MSFC) (Table 2), although these levels were similar to controls both in MS [141] and early MS patients [54]. CSF NAA levels were shown to be lower in SPMS patients compared to RR and PPMS cases [54, 141] (Table 4). Teunissen et al. reported a decrease in CSF NAA levels as the disease progressed, therefore possibly reflecting the accumulation of axonal degeneration in a later MS stage [54]. One study has shown CSF NAA concentrations to correlate with age [54].

### 3.9. Apolipoprotein E

Apolipoprotein E (Apo-E) is mostly produced by astrocytes in the CNS, but it is also found in neurons [21]. There are three different alleles of the human Apo-E gene coding for the three isoforms: *ε*2, *ε*3, and *ε*4 [145]. Apo-E is generally involved in lipid transport and cholesterol homeostasis [21]. However, its functions within the CNS are not so clear and might include immunomodulation, a protective role against oxidative stress [146], a role in maintaining the BBB integrity [147], a role in myelin lipid metabolism [148], and a potential role in neurorepair [21]. As there is a limited permeability of Apo- E across the BBB, Apo- E changes in CSF might dominantly result from a reduction of its local synthesis and secretion by brain tissue [146, 149].

 Several studies have indicated that Apo-E *ε*4 allele might be associated with MS, although evidence is still not sufficient enough [146, 150]. Although plausible [151], the association between Apo-E and MS course and disease severity remains controversial [152]. Apo-E genotypes seem not to influence the development of MS, but Apo-E *ε*4 allele might predispose carriers with MS to a faster disease progression [153, 154]. In line with this, lower levels of NAA in MS patients with the Apo-E *ε*4 allele have been demonstrated by MRS [155]. It has been recently suggested that Apo-E polymorphism may interact with cigarette smoking in promoting MS progression [156]. Although some authors have shown an association between Apo-E *ε*4 and cognitive impairment in MS patients [157], the others could not confirm this finding [158].

However, it was suggested that the decreased CSF Apo-E concentration in MS patients occurs independent of the Apo-E genotype [159]. In one study the plasma concentration of Apo-E was significantly lower in MS patients than in healthy controls although its CSF concentrations were similar in these two groups [148]. Rifai et al. have shown higher CSF/serum Apo-E index in RRMS in remission compared to controls [160], Chiasserini et al. demonstrated an increased CSF expression of an Apo-E isoform in RRMS compared to CIS patients or controls [161], whereas Gaillard et al. [159] found lower concentrations of both CSF Apo-E and intrathecal Apo-E in MS patients than in controls (Table 5). No correlation of Apo-E in serum or CSF with age or clinical course was found [159].

### 3.10. Neuron-Specific Enolase

Enolase is a glycolytic enzyme (2-phospho-D-glycerate hydrolase), which exists in three isoforms: **α**-enolase, which is ubiquitous, **β**-enolase, which is predominant in muscle, and **γ**-enolase (neuron-specific enolase, NSE), which is found in neurons, glial, and neuroendocrine cells [84]. NSE was shown to be an indicator of the acute neuro-destructive disorders [162], but its levels are usually normal in MS patients [162] and no clear difference in the NSE levels has been observed between MS patients and controls in CSF [64] or serum [142], or between patients with RR and SPMS in CSF [144] (Table 4). NSE concentration in CSF and serum was shown to be decreased in CIS patients when compared to the control group, potentially indicating a reduced neuronal metabolic activity at the early stage of the disease [105]. Interestingly, Forooghian et al. demonstrated a higher T-cell responses against NSE in the *peripheral blood* mononuclear cells of MS patients than in controls [84]. One study which followed CSF NSE levels in MS patients treated with intrathecal triamcinolone administration did not show any changes in its levels following treatment [163]. A recent study examined plasma NSE levels in MS patients over a five-year period and found a strong inverse relationship between serum NSE levels and disease progression [143] as expressed through EDSS and MSSS score changes, thus potentially reflecting a reduced metabolic activity secondary to axonal loss. In two other studies no correlation of CSF [64, 105] or serum [105] NSE levels were found with EDSS (Table 2). Age-related changes of NSE in CSF with an increase of 1% per year have been reported [164], but in some other studies CSF NSE levels were independent of gender, age [64, 165], and disease duration [64].

### 3.11. Growth-Associated Protein 43

Growth-associated protein 43 (GAP-43), also known as B-50 or neuromodulin, is a marker associated with growth cones, synaptic plasticity, and synaptic regeneration [166]. It is a calmodulin-binding protein being attached to the cytoplasmic site of the axonal membrane, involved in neurotransmitter release [167] which also stimulates neurite outgrowth [166]. A decreased GAP-43 expression was found in postmortem white matter MS lesions, independent of the lesion stage, whereas increased or unaltered expression was detected in remyelinated lesions and was found unchanged in grey matter lesions [167]. 

In a recent study, CSF GAP-43 levels were comparable among RR/SP and PPMS subtypes and controls and GAP-43 was not detected in serum [167] (Table 5). A tendency towards a negative correlation between CSF GAP-43 levels and EDSS was found, but CSF GAP 43 levels positively correlated with MRI measures of atrophy [167] (Table 2). Moreover, a positive correlation was observed between CSF NAA and GAP-43 levels [141]. No significant correlation was reported between CSF GAP-43 levels and age, gender, and disease duration [167].

### 3.12. 24S-Hydroxycholesterol

Cholesterol plays a crucial structural role in the brain [168] being the main lipid of neuronal membranes [21]. For maintenance of brain cholesterol homeostasis [168], cholesterol is converted into its more polar metabolite cerebrosterol (24S-hydroxycholesterol, 24S-OH-chol) by the CNS-specific cytochrome P450 enzyme CYP46 [169]. Virtually all of the cerebrosterol in the peripheral circulation is CNS derived and its blood levels are assumed to reflect the relation between cholesterol CNS production caused by demyelination or neurodegeneration and hepatic clearance [170, 177, 178]. The level of 24S-OH-chol highly correlated with total cholesterol and the ratio between 24S-OH-chol and cholesterol is assumed to be a better marker for the cerebral production than the absolute cerebrosterol concentration [171]. The majority of daily efflux of this oxysterol from the brain to the circulation apparently occurs as a direct transport across the BBB and less than 1% of the total flux of 24S-OH-chol from the brain occurs via CSF [168], which might cause the lack of correlation between CSF and plasma levels of this metabolite [171]. 

The higher CSF levels of 24S-OH-chol were shown in patients with gadolinium-enhancing MRI lesions, indicating the pronounced release of the 24S-OH-chol from damaged cells during CNS inflammation [171, 173] (Table 2). Moreover, patients with a defective BBB were found to have markedly increased absolute levels of 24-OH-chol in CSF [179]. Karrenbauer et al. [169] demonstrated a negative correlation between the cerebrosterol/cholesterol ratio in plasma and volume of T_2_-weighted MRI lesions, whereas a significant inverse relation between the EDSS score and plasma cholesterol-related levels of 24S-OH-chol was found in the other study [171]. Teunissen et al. [172] showed the reduction in serum 24S-OH-chol concentrations to be most pronounced in the PP clinical subtype (Table 5). Leoni et al. found a tendency to increased plasma levels of 24S-OH-chol in younger patients with high levels in the 3rd and 4th decades of life, and significantly lower levels in older MS patients aged 51–70 years than in healthy age-matched controls [171]. There seems to be no gender influence on plasma levels of 24S-OH-chol or the ratio between cerebrosterol/cholesterol [171] and no correlation of the latter with disease duration was reported to date [172].

### 3.13. Protein 14-3-3

14-3-3 family proteins are ubiquitous, highly conserved proteins with the highest concentrations in brain [162, 180] and within CNS are constitutively expressed in neurons and glia both in cytoplasmic and nuclear regions [181] with small amounts bound to synaptic membranes [162]. A growing body of evidence indicates that it might act as a novel type of molecular chaperone which interacts with key molecules involved in cell differentiation, proliferation, and transformation [182], and recent data suggested its antiapoptotic effects [183, 184]. The detection of 14-3-3 protein in the CSF is highly sensitive for *in vivo *diagnosis of Creutzfeldt-Jakob disease [185], but this protein, in the CSF, could be also detected in some other prion-unrelated conditions associated with CNS tissue damage [175, 181, 186]. 

The 14-3-3 protein is more frequently detectable in the CSF of MS or CIS patients than in controls although in such cases it is present in a small subgroup of patients [108, 174]. However, in the study of Colucci et al. [140] it was more frequently positive than previously reported (Table 5). The detection of the 14-3-3 protein in the CSF of CIS patients was shown to be an independent predictor of short-term conversion to CDMS [175, 176] (Table 2). Moreover, the 14-3-3 positive group had a significantly higher relapse rate and a higher frequency of patients with EDSS ≥2.0 after a median followup of 33.4 months [176], which confirmed previous results by the same authors [175]. In some studies, 14-3-3 protein positivity in MS patients was associated with a more severe disability [140, 187] and the rate of disease progression during a mean of 10-month clinical followup [140], but was also shown to be a potential predictor of permanent neurological disability after an episode of the acute transverse myelitis [188]. However, the latter was not shown in two other studies [115, 174]. The presence of 14-3-3 reactivity was not shown to prevail in MS clinical subgroups [140] and seems not to correlate with age [108] or disease duration [140].

### 3.14. Proteomics Research

Recently, a rapid development of proteomic approaches refocused biomarker research interest to the use of novel methods in the discovery of potential MS-specific biomarkers in biological fluids and especially in the CSF [189, 190]. Among the wide range of proteins that have been found to be exclusively present in the CSF of MS patients [125, 189, 191–194], only some of them are expressed on neurons (contactin-1, neurofascin, neurotrimin, and chromogranins/secretogranins) [193–195]. It was recently shown that contactin-2 was recognised by both autoantibodies and Th1/Th17 T-cells in MS patients [36, 37] and neurofascin-specific autoantibodies were identified in MS patients [196]. However, there is a range of neuroaxonal proteins which still need to be studied in CIS/MS patients although some of them have been investigated in animal models [197].

## 4. Summary and Future Directives

Based on the majority of available results, the increased levels of the CSF NF-L or NF-H seem to be present even at early MS phases, a scenario which continues during the entire course of the disease and correlates with different measures of disability; the increased levels of these markers seem to be more pronounced in active disease states and have a potential value in an effort to predict conversion to CDMS after a first CIS episode, estimate future progression and disability, but their value for the prediction of treatment response still has to be investigated, most importantly in early MS patients. 

Although the presence of anti-NF antibodies could, in part, be an epiphenomenon of the disease, the elevated levels of these antibodies in progressive disease and correlations with disease duration and disability indicate a rise in antibodies induced by axonal destruction, but also a possible pathogenic role of these antibodies in promoting axonal damage and disease progression. This indicates that serum and/or CSF anti-NF-L, NF-M, and NF-H antibodies might be a potential a marker of CNS tissue damage in MS, but their potential predictive value for the future disease course, disability, disease progression, and treatment response needs to be investigated. 

CSF levels of actin and tubulin seem to be elevated in progressive MS and correlate with disability, but their levels in early MS patients, as well as the potential predictive value have been underreported to date. 

It is possible that elevated CSF t-tau levels are present from early MS phases and increase in clinically/MRI active disease phases; although its potential correlation with ongoing disease progression has been indicated, the reports related to this molecule so far have been quite contradictory and its validity as a biomarker needs to be further studied both in blood and CSF. 

CSF and blood levels of APP-derived proteins seem not to be reliable markers of disease activity or progression since their levels are largely dependent on complex regulatory metabolic processes which could be highly variable in a complex and heterogenous disease such as MS. 

A correlation of CSF NAA levels with disability measures even in CIS patients suggests the potential clinical relevance of this molecule as a biomarker that should be further investigated.

NSE and Apo-E levels in CSF/blood are not consistently abnormal in MS patients and their relation to neuroaxonal damage is complicated since the expression of both molecules is not limited to neurons. 

CSF/blood levels of GAP-43 were investigated to date in a paucity of studies and some preliminary results might indicate the need for further investigations of this molecule as of the potential biomarker of disease progression and disability. 

Serum 24S-OH-chol levels seem to be as reliable as levels in CSF to estimate neuronal membrane status. Some reports indicated its correlation with disability and MS disease activity and thus the validity of this molecule as a biomarker should be further investigated. 

The 14-3-3 family proteins could be potentially related to CIS conversion to MS, disability, and its progression, but this still has to be further confirmed. Astrocyte-derived 14-3-3 protein could complicate the relation of CSF/blood levels of 14-3-3 protein only to neuroaxonal status in MS. 

Additionally, it would be desirable to systematically compare the proteome profiles of MS subgroups at a defined disease stages and in large cohorts in order to identify proteins which are consistently present in the CSF at a certain disease phase in a given subgroup, a task which is still facing a lot of obstacles. 

So far, the abovementioned markers have been investigated in the light of their significance to reflect the presence and the extent of neuroaxonal damage in CIS/MS patients. Since each of them could be related to different structural levels of neuroaxonal loss of integrity, the combined evaluation of these markers could be more informative on the ongoing neurodegenerative process [54]. Moreover, the relevance of the single biomarker has to be judged in the light of disease stage and/or disease activity since biomarker levels could show temporal dynamics that correlates with the dynamics of the MS natural course [54, 198].

The results of the biomarker studies could have been influenced by small study sizes, cross-sectional designs, and insufficient followup to allow meaningful conclusions [199]. Biomarker studies in MS neurodegeneration have been conducted in a variable patient population, varying from a few [68] to over a hundred patients included [108, 171]. Moreover, followup in most of these studies was up to three years [54, 62, 113, 140, 175, 176] which could allow only tentative conclusions on biomarker's long-term prognostic significance. The differences in preanalytical processes and different assay sensitivities could also cause contradictory results in biomarker studies [199]. The comparable results were shown in several studies that used the same NF-H Enzyme-Linked Immunosorbent Assay (ELISA) method [62, 70–72]. However, a poor interlaboratory coefficient of variation in a recent multicenter NF-L ELISA validation study has been shown, mainly due to the lack of preparation of accurate and consistent protein standards [200]. Therefore, a standardization of body fluid sampling and storage [201], as well as the use of the standardized and validated assay procedures [23, 200], are needed. 

Since none of the potential CSF/blood biomarkers studied so far fulfils all necessary criteria for a surrogate biomarker [22] there is an ongoing need for further biomarker studies, especially those aiming to predict future disease course, disability, and/or treatment response at the early MS stage.

##  Conflict of Interests 

There is no conflict of interests to declare.

## Figures and Tables

**Table 1:** Neurofilament subunits in the cerebrospinal fluid (CSF) and blood of patients with multiple sclerosis (MS) and/or clinically isolated syndrome suggestive of MS (CIS).

Biomarker (subtype)	Body fluid	Immunoassay	Number of patients	Main findings	REF
NF-L* (cytoplasmic) *	CSF	ELISA	60 MS (RR)	RR ↑↑ HCo	[61]
	CSF	Dot-blot	35 MS (RR/SP/PP)	MS ↑↑ OIND or NIND, PP/SP ↑↑ RR	[63]
	CSF	ELISA	66 MS (RR/SP)	RR/SP ↑↑ HCo	[64]
	CSF	ELISA	99 MS (RR/SP/PP/PR)	RR/SP/PP ↑↑ NHCo, SP the highest	[65]
	CSF	ELISA	51 MS (RR/SP/PP)	not detected	[66]
	CSF	ELISA	47 MS	MS ↑↑ healthy siblings or HCo	[67]
	CSF	ELISA	76 MS (RR/SP/PP) + 38 CIS	CIS+MS ↑↑ NIND+OIND + NHCo	[54]
	CSF	ELISA	5 MS (RR)	MS ↑↑ NHCo	[68]

NF-M* (cytoplasmic) *	CSF/PL/SE	not investigated	—	—	—

NF-H* (cytoplasmic) *	CSF	ELISA	38 MS (RR) + 52 CIS	NF-H^SM135^ in CIS ↑↑ NHCo	[25]
	CSF	ELISA	41 ON	NF-H^SM134^ and NF-H^SM134/SM135^ in ON ↑↑ OND	[69]
	CSF	ELISA	51 MS (RR/SP/PP)	NF-H^SM135^ RR *↔* SP *↔* PP	[66]
	CSF	ELISA	34 MS (RR/SP/PP)	NF-H^SM134^ in PP/SP ↑↑ RR, NF-H^SM135^ in SP/PP ↑↑ RR	[62]
	CSF	ECL	95 MS	MS ↑↑ NHCo	[23]
	CSF	ELISA	24 MS (RR/SP/PP)	NF-H^SM135^ in SP ↑ RR	[70]
	PL	ELISA	18 ON	NF-H^SM135^ in ON ↑↑ HCo	[71]
	CSF	ELISA	34 MS (RR)	NF-H^SM135^ in MS ↑↑ NHCo	[72]
	CSF	ELISA	76 MS (RR/SP/PP) + 38 CIS	NF-H^SM135^ in MS+CIS ↑↑ NHCo + NIND + OIND	[54]

REF: reference; NF-L: neurofilament-light chain; NF-M: neurofilament-intermediate chain; NF-H: neurofilament-heavy chain; PL: plasma; SE: serum; ELISA: Enzyme-Linked Immunosorbent Assay; ECL: Electrochemiluminescence-based solid-phase sandwich immunoassay; RR: relapsing-remitting MS; SP: secondary-progressive MS; PP: primary-progressive MS; ON: optic neuritis; NF-H^SM135^: NF-H phosphorylated form; NF-H^SM134^: NF-H hyperphosphorylated form; ↑↑ significantly higher than; ↑ higher than; *↔* no difference between; HCo: healthy controls; OIND: other inflammatory neurological diseases; NIND: noninflammatory neurological disorders; NHCo: neurologically healthy controls; OND: other neurological diseases.

**Table 2:** Biomarkers of neuroaxonal damage in patients with multiple sclerosis (MS) and/or clinically isolated syndrome suggestive of MS (CIS) in relation to clinical parameters.

Biomarker	Correlation with disability*	Correlation with disease activity*	Prediction of CIS conversion to CDMS*	Prediction of future disease course*	Prediction of future disability*	Prediction of treatment response*
NF-L	4 (308) + 2 (117) −	4 (339) +	1 (38) +	1 (95) +	3 (308) +	—
Anti-NF-L	3 (180) + 2 (181) −	—	—	—	—	—
Anti-NF-M	1 (47) + 1 (49) −	—	—	—	—	—
NF-H	5 (256) + 2 (81) −	3 (254) + 1 (30) −	1 (52) +	1 (34) +	3 (86) +	1 (30)** + 1 (32) +
Anti-NF-H	1 (67) + 1 (51) −	—	—	—	—	—
Tubulin	1 (35) +	—	—	—	—	—
Antitubulin	1 (67) + 2 (81) −	—	—	—	—	—
Actin	1 (35) +	—	—	—	—	—
Tau	1 (90) + 4 (218 ) −	3 (179) + 1 (90) −	1 (52)** + 1 (53) −	1 (32) +	1 (32) + 1 (53) −	—
Amyloid *β*42	1 (21) −	1(21) +	—	—	—	—
BACE1	—	—	—	—	1 (100) −	—
NAA	2 (160) +	—	—	—	—	—
Apo-E	—	—	—	—	—	—
NSE	1 (64) + 2 (87) −	—	—	—	—	—
GAP-43	1 (49)** +	—	—	—	—	—
24S-OH-chol	1 (118) +	2 (206) +	—	—	—	—
14-3-3	2 (82) +	1 (38) +	2 (123) +	—	2 (101) +	—

*Number of positive (+) or negative (−) studies with total number of patients included (in brackets); **a tendency; CDMS: clinically definite MS; NF-L: neurofilament-light chain; Anti-NF-L: antibodies to NF-L; Anti-NF-M: antibodies to neurofilament-intermediate chain; NF-H: neurofilament-heavy chain; Anti-NF-H: antibodies to NF-H; BACE1: *β*-site amyloid precursor protein-cleaving enzyme 1; NAA: N-acetylaspartate; Apo-E: apolipoprotein-E; NSE: neuron-specific enolase; GAP-43: growth-associated protein 43; 24S-OH-chol: 24S-hydroxycholesterol.

**Table 3:** Parameters of humoral and cellular response to markers of neuroaxonal damage in the cerebrospinal fluid (CSF) and blood of patients with multiple sclerosis (MS) and clinically isolated syndrome suggestive of MS (CIS).

Biomarker	Body fluid	Immunoassay	Number of patients	Main findings	REF
Anti-NF-L	CSF/SE	ELISA (IgG)	67 MS (RR/SP/PP)	CSF/SE index in PP or SP ↑↑ RR or OIND/NIND/NHCo	[74]
	CSF/SE	ELISA (IgM, IgG)	58 MS (RR/SP/PP) + 8 CIS	specific IgG-index in MS ↑↑ CD	[79]
	CSF/SE	ELISA (IgG)	51 MS (RR/SP/PP)	CSF/SE index correlated with brain atrophy, RR *↔* SP *↔* PP	[66]
	CSF/SE	ELISA (IgG)	130 MS (RR/SP/PP)	serum antibody levels in PP ↑↑ OND or HCo	[80]

Anti-NF-M	CSF/SE	ELISA (IgG)	47 MS (RR/SP/PP)	significant correlation with anti-NF-L and antitubulin IgG in serum and CSF	[81]
	CSF/SE	ELISA (IgG, IgM)	49 MS (RR/SP/PP)	IgM and IgG specific indices in MS subgroups ↑↑ CD or CN	[82]

Anti-NF-H	CSF/SE	ELISA (IgG)	51 MS (RR/SP/PP)	RR *↔* SP *↔* PP	[66]
	CSF/SE	ELISA (IgG)	67 MS (RR/SP/PP)	MS *↔* OIND/NIND/NHCo	[74]

Antitubulin	CSF/SE	ELISA (IgG)	67 MS (RR/SP/PP)	MS *↔* OIND/NIND/NHCo	[74]
	CSF/SE	ELISA (IgG)	47 MS (RR/SP/PP)	significant correlation with anti-NF-L and anti-NF-M IgG in serum and CSF	[81]
	CSF/SE	ELISA (IgG)	29 MS (RR/SP/PP) + 5CIS	CSF levels in MS+CIS ↑↑ CN	[83]

Anti-NSE T-cell response	PBMC	T-cell Proliferation Assay	35 MS	prevalence of response in MS ↑↑ HCo	[84]

REF: reference; Anti-NF-L: antibodies to neurofilament-light chain; anti-NF-M: antibodies to neurofilament-intermediate chain; anti-NF-H: antibodies to neurofilament-heavy chain; NSE: neuron-specific enolase; SE: serum; PBMC: peripheral blood mononuclear cells; ELISA: Enzyme-Linked Immunosorbent Assay; IgG: immunoglobulin G, IgM: immunoglobulin M; RR: relapsing-remitting MS; SP: secondary-progressive MS; PP: primary-progressive MS; ↑↑ significantly higher than; *↔* no difference between; OIND: other inflammatory neurological diseases; NIND: noninflammatory neurological disorders; NHCo: neurologically healthy controls; CD: miscellaneous neurological diseases; OND: other neurological diseases; HCo: healthy controls, CN: normal controls (vertigo, headache, psychogenic syndrome, and fatigue).

**Table 4:** Cytoplasmic, non-neurofilament biomarkers of neuroaxonal damage in the cerebrospinal fluid (CSF) and blood of patients with multiple sclerosis (MS) and/or clinically isolated syndrome suggestive of MS (CIS).

Biomarker	Body fluid	Immunoassay	Number of patients	Main findings	REF
Tubulin	CSF	Dot-blot	35 MS (RR/SP/PP)	MS ↑↑ OIND or NIND, PP/SP ↑↑ RR	[63]

Actin	CSF	Dot-blot	35 MS (RR/SP/PP)	MS ↑↑ OIND or NIND, PP/SP ↑↑ RR	[63]

Gelsolin	CSF/PL	Western blot	56 MS	PL levels in MS ↓↓ Co*, CSF levels in MS *↔* Co*	[96]

Tau	CSF	ELISA (t-t)	38 MS (RR) + 52 CIS	CIS ↑↑ NHCo	[25]
	CSF	ELISA (t-t)	45 MS (RR/SP/PP)	MS ↑↑ OIND + NIND, SP *↔* RR *↔* PP	[110]
	CSF	ELISA (t-t, p-t)	42 RRMS + 18 CIS	t-tau and p-tau in MS+CIS↑↑ NHCo, t-tau in CIS ↑↑ NHCo	[109]
	CSF	ELISA (t-t)	38 MS (RR/SP/PP) + 12 CIS	MS+CIS*↔* NHCo	[104]
	CSF	ELISA (t-t)	76 MS (RR/SP/PP) + 38 CIS	MS/CIS *↔* NHCo	[54]
	CSF	ELISA (t-t)	114 MS (RR/SP/PP)	MS ↑↑ NIND	[108]
	CSF	ELISA (t-t)	52 MS (RR/SP/PP) + 50 CIS	MS+CIS ↑↑ NHCo, the highest in CIS	[103]
	CSF	ELISA (t-t)	20 MS (RR/progressive MS)	MS *↔* NHCo	[111]
	CSF	ELISA (t-t)	36 MS (RR/SP/PP)	MS ↑↑ NHCo	[107]
	CSF	ELISA (t-t, p-t)	25 RRMS	MS *↔* OIND or NIND	[112]
	CSF/SE	ELISA (t-t, p-t)	21 CIS	CIS *↔* Co**	[105]
	CSF	ELISA (t-t, p-t)	14 MS + 9 CIS	MS *↔* CIS *↔* NHCo	[106]
	CSF	ELISA (t-t)	43 MS (RR/SP/PP/PR) + 20 CIS	MS *↔* CIS *↔* NHCo+OND	[140]

Amyloid *β*42	CSF/SE	ELISA	21 CIS	CIS *↔* Co**	[105]
Amyloid *β*42	CSF	ELISA	14 MS + 9 CIS	MS ↑↑NHCo	[106]
Amyloid *β*42	CSF	xMAP	100 MS (RR/SP/PP)	MS ↓↓ NHCo	[122]
*α*-sAPP	CSF	Multiplex Assay	100 MS (RR/SP/PP)	MS ↓↓ NHCo	[122]
*β*-sAPP	CSF	Multiplex Assay	100 MS (RR/SP/PP)	MS ↓↓ NHCo	[122]

Bri2-23	CSF	SELDI-TOF	40 MS (SP/PP)	MS ↓ OND	[125]

NAA	CSF	GC-MS	76 MS (RR/SP/PP) + 38 CIS	SP ↓↓ RR/CIS, CIS *↔* RR *↔* NHCo	[54]
	CSF	GC-MS	46 MS (RR/SP/PP)	MS *↔* OND, SP ↓↓ RR	[141]

NSE	CSF	Luminescence	66 MS (RR/SP)	MS *↔* HCo	[64]
	SE	RIA	21 MS	levels within normal range	[142]
	PL	Luminescence	64 MS (RR/SP/PP)	progressive MS ↓ RR	[143]
	CSF/SE	ELISA	21 CIS	CIS ↓ Co**	[105]
	CSF	Immunoluminometry	33MS (RR/SP)	RR *↔* SP	[144]

REF: reference; APP: amyloid-precursor protein; NAA: N-acetylaspartate; NSE: neuron-specific enolase; SE: serum; PL: plasma; ELISA: Enzyme-Linked Immunosorbent Assay; t-t: total tau protein, p-t: abnormally phosphorylated tau protein; xMAP: xMap Bead-based immunoassay; SELDI-TOF: Surface-Enhanced Laser Desorption/Ionization Time-Of-Flight; GC-MS: stable isotope dilution gas chromatography-mass spectrometry; RIA: radioimmunoassay; RR: relapsing-remitting MS; SP: secondary-progressive MS; PP: primary-progressive MS; ↑↑ significantly higher than; ↓↓ significantly lower than; ↓ lower than; *↔* no difference between; OIND: other inflammatory neurological diseases; NIND: noninflammatory neurological disorders; Co: controls with *idiopathic headache, Bell's palsy and ischialgia or **idiopathic headache and migraine; NHCo: neurologically healthy controls; OND: other neurological diseases; HCo: healthy controls.

**Table 5:** Membrane and nuclear biomarkers of neuroaxonal damage in the cerebrospinal fluid (CSF) and blood of patients with multiple sclerosis (MS) and/or clinically isolated syndrome suggestive of MS (CIS).

Biomarker (subtype)	Body fluid	Immunoassay	Number of patients	Main findings	REF
BACE1 * (membrane) *	CSF	Enzymatic	100 MS (RR/SP/PP)	MS slightly↓NHCo	[122]

Apo- E * (membrane) *	CSF/SE	Immunofluorometry	34 MS	CSF levels in MS ↓↓ NHCo	[159]
	CSF	2DE-MS	10 RRMS + 11 CIS	one isoform expression in RR ↑↑ CIS or NHCo	[161]
	CSF/SE	Immunoturbidimetry	33 MS (RR)	CSF/serum index in MS in remission ↑↑ NHCo	[160]

GAP-43 * (membrane) *	CSF/SE	xMap Bead –based	49 MS (RR/SP/PP)	CSF in RR *↔* SP *↔* PP *↔* OIND + NIND + HCo, not detected in serum	[167]
	CSF	xMap Bead –based	44 MS (RR/SP/PP)	significant positive correlation with NAA levels	[141]

24S-OH-chol * (membrane) *	PL	IDMS	46 MS (RR/PP)	negative correlation with T2 lesion volume	[169]
	PL	IDMS	11 MS	MS *↔* HCo	[170]
	CSF/PL	IDMS	118 MS (RR/SP/PP)	PL levels in older MS ↓↓ HCo	[171]
	SE	IDMS	60 MS (RR/SP/PP)	PP or older RR ↓ HCo	[172]
	CSF/PL	IDMS	88 MS	PL levels in MS *↔* Co	[173]

Protein 14-3-3 * (cytoplasmic, nuclear, membrane) *	CSF	Western blot	22 MS (RR/SP/PP) + 15 ATM	detected in about 8% RR/ATM patients	[174]
	CSF	Immunoblot	38 CIS	detected in 13% CIS patients	[175]
	CSF/SE	Immunoblot	21 CIS	detected in 1/21 patient	[105]
	CSF	ELISA	114 MS (RR/SP/PP)	detected in 22% MS patients, not detected in HCo	[108]
	CSF	Immunoblot	43 MS (RR/SP/PP/PR) + 20 CIS	detected in 38% CIS/MS, similar in MS subgroups	[140]
	CSF	Immunoblot	85 CIS	detected in 8.2 % CIS patients	[176]

REF: reference; BACE1: *β*-site amyloid precursor protein-cleaving enzyme 1; Apo-E: Apolipoprotein-E; GAP-43: growth-associated protein-43; 24S-OH-chol: 24S-hydroxycholesterol; SE: serum; PL: plasma; Enzymatic: Enzymatic solution-based assay; 2DE-MS: two-dimensional electrophoresis-mass spectrometry; IDMS: isotope-dilution mass spectrometry; ELISA: Enzyme-Linked Immunosorbent Assay; RR: relapsing-remitting MS; SP: secondary-progressive MS; PP: primary-progressive MS; ATM: acute transverse myelitis; NAA: N-acetylaspartate; ↓ lower than; ↓↓ significantly lower than; ↑↑ significantly higher than; *↔* no difference between; NHCo: neurologically healthy controls; OIND: other inflammatory neurological diseases; NIND: noninflammatory neurological disorders; HCo: healthy controls; Co: controls with idiopathic headache.
